# Chloridobis[2-(1,5-dimethyl-1*H*-pyrazol-3-yl-κ*N* ^2^)-1-methyl-1*H*-imidazole-κ*N* ^3^]copper(II) chloride methanol hemisolvate tetra­hydrate

**DOI:** 10.1107/S1600536810000048

**Published:** 2010-01-09

**Authors:** Lhoussaine El Ghayati, El Mostafa Tjiou, Lahcen El Ammari

**Affiliations:** a Laboratoire de Chimie Organique Hétérocyclique, Pôle de Compétences, Pharmacochimie, Avenue Ibn Battouta, BP 1014, Faculté des Sciences, Université Mohammed V-Agdal, Rabat, Morocco; b Laboratoire de Chimie du Solide Appliquée, Faculté des Sciences, Université Mohammed V-Agdal, Avenue Ibn Battouta, BP 1014, Rabat, Morocco

## Abstract

In the title compound, [CuCl(C_9_H_12_N_4_)_2_]Cl·0.5CH_3_OH·4H_2_O, the Cu^II^ ion adopts a distorted trigonal-bipyramidal coordination arising from two bidentate ligands and a Cl^−^ anion. The two heterocyclic ligands are planar with dihedral angles of 3.4 (1) and 0.7 (1)° between the pyrazole and imidazole rings. In the crystal, water mol­ecules and uncoordinated chloride anions form an O—H⋯Cl and O—H⋯O hydrogen-bonded sheet parallel to (100) which lies between two layers of complex mol­ecules. The packing is further stabilized by C—H⋯Cl and C—H⋯O hydrogen bonds. The methanol solvent mol­ecule is disordered across a centre of inversion.

## Related literature

For applications of transition metal complexes with biheterocyclic ligands, see: Allen & Wilson (1963 ▶); El-Khawass & Bistawroos (1990 ▶); Pearson (1975 ▶); Trofimenko (1993 ▶); Tsuboi *et al.* (1994 ▶); Hartfiel *et al.* (1993 ▶). For the preparation of biheterocyclic ligands, see: Tjiou *et al.* (1989 ▶); Bouhaddioui (1993 ▶).
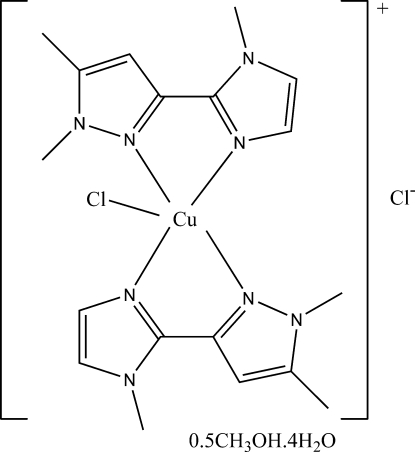


## Experimental

### 

#### Crystal data

[CuCl(C_9_H_12_N_4_)_2_]Cl·0.5CH_4_O·4H_2_O
*M* *_r_* = 574.98
Monoclinic, 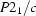

*a* = 12.5213 (3) Å
*b* = 15.5386 (4) Å
*c* = 14.1806 (4) Å
β = 100.883 (1)°
*V* = 2709.40 (12) Å^3^
*Z* = 4
Mo *K*α radiation
μ = 1.04 mm^−1^
*T* = 298 K
0.44 × 0.33 × 0.19 mm

#### Data collection

Bruker X8 APEXII area-detector diffractometer
Absorption correction: multi-scan (*SADABS*; Bruker, 2005 ▶) *T*_min_ = 0.668, *T*_max_ = 0.820
47954 measured reflections
7884 independent reflections
5480 reflections with *I* > 2σ(*I*)
*R*_int_ = 0.029

#### Refinement

*R*[*F* ^2^ > 2σ(*F* ^2^)] = 0.038
*wR*(*F* ^2^) = 0.124
*S* = 1.01
7884 reflections
323 parameters
H-atom parameters constrained
Δρ_max_ = 0.39 e Å^−3^
Δρ_min_ = −0.26 e Å^−3^

### 

Data collection: *APEX2* (Bruker, 2005 ▶); cell refinement: *SAINT* (Bruker, 2005 ▶); data reduction: *SAINT*; program(s) used to solve structure: *SHELXS97* (Sheldrick, 2008 ▶); program(s) used to refine structure: *SHELXL97* (Sheldrick, 2008 ▶); molecular graphics: *ORTEP-3 for Windows* (Farrugia,1997 ▶) and *PLATON* (Spek, 2009 ▶); software used to prepare material for publication: *WinGX* (Farrugia, 1999 ▶).

## Supplementary Material

Crystal structure: contains datablocks I, global. DOI: 10.1107/S1600536810000048/ci2996sup1.cif

Structure factors: contains datablocks I. DOI: 10.1107/S1600536810000048/ci2996Isup2.hkl

Additional supplementary materials:  crystallographic information; 3D view; checkCIF report

## Figures and Tables

**Table 1:** Selected bond lengths (Å)

Cu1—N1	1.9531 (17)
Cu1—N5	1.9545 (17)
Cu1—N4	2.2161 (14)
Cu1—N8	2.2415 (14)
Cu1—Cl1	2.2739 (6)

**Table 2:** Hydrogen-bond geometry (Å, °)

*D*—H⋯*A*	*D*—H	H⋯*A*	*D*⋯*A*	*D*—H⋯*A*
O1—H1*A*⋯Cl2^i^	0.85	2.33	3.162 (2)	167
O1—H1*B*⋯Cl2	0.84	2.34	3.186 (2)	175
O2—H2*A*⋯Cl2	0.83	2.39	3.205 (3)	165
O3—H3*B*⋯Cl2^ii^	0.85	2.38	3.234 (3)	174
O4—H4*A*⋯O1	0.84	1.98	2.793 (3)	165
O4—H4*B*⋯O2^iii^	0.83	1.89	2.706 (4)	165
C11—H11⋯Cl2^iv^	0.93	2.75	3.592 (2)	151
C18—H18*C*⋯Cl1^v^	0.96	2.76	3.708 (3)	177

Symmetry codes: (i) 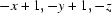; (ii) 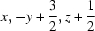; (iii) 
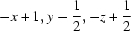
; (iv) 
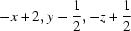
; (v) 
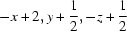
.

**Fig. 1.**
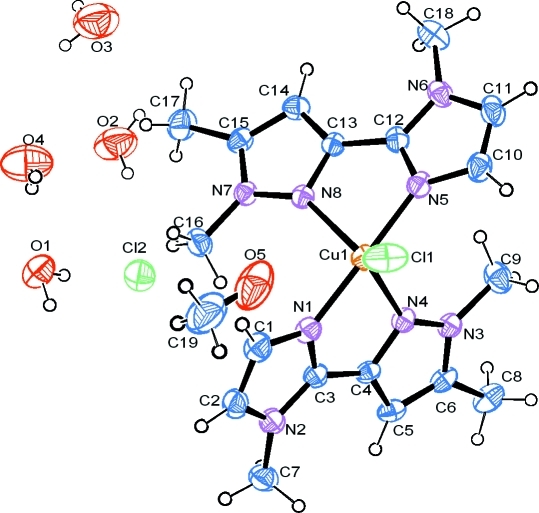
The asymmetric unit of the title compound, with the atom-labeling scheme. Displacement ellipsoids are drawn at the 50% probability level. H atoms are represented as small spheres of arbitrary radii.

**Fig. 2.**
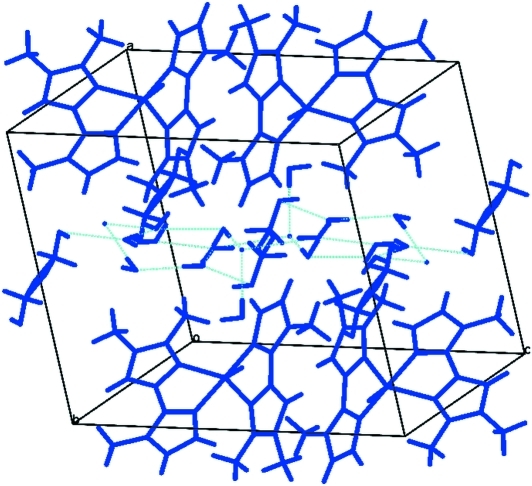
Packing diagram showing hydrogen-bonded (dashed lines) layer of solvent molecules between the complex molecules.

## supplementary crystallographic information

### Comment

The ability of biheterocycles to form stable and biochemically interesting complexes, with transition metals has prompted several researchers to test them in several areas: medicine (El-Khawass & Bistawroos, 1990, Trofimenko, 1993), agriculture (Tsuboi *et al.*, 1994, Hartfiel *et al.*, 1993) and the photography industry (Allen & Wilson, 1963; Pearson, 1975). To contribute to the understanding of interaction of these heterocyclic compounds with transition metals, we have studied a copper complex of a biheterocycle prepared by Tjiou *et al.* (1989) and methylated using phase transfer catalysis process (Bouhaddioui, 1993).

The Cu^II^ ion adopts a distorted trigonal bipyramidal coordination arising from two bidentate ligands and a Cl^-^ anion (Fig. 1). The axial positions are occupied by N1 and N5 [N1—Cu1—N5 = 173.03 (7)°], while atoms Cu1, Cl1, N4 and N8 lie in the equatorial plane [N4—Cu1—Cl1 = 128.60 (4)°, N8—Cu1—Cl1 = 132.50 (4)° and N4—Cu1—N8 = 98.90 (6)°]. The two organic ligands are almost planar; the dihedral angle between N1/C1/C2/N2/C3 and N3/N4/C4-C6 planes is 3.4 (1)° and that between N5/C10/C11/N6/C12 and N7/N8/C13-C15 planes is 0.7 (1)°.

In the crystal, the water molecules and uncoordinated chloride ions form a O—H···Cl and O—H···O hydrogen-bonded sheet parallel to the (100) and it lies between two layers of complex molecules. The packing is further stabilized by C—H···Cl and C—H···O hydrogen bonds (Table 2 and Fig.2).

### Experimental

The title compound was synthesized by mixing a solution of biheterocycle in methanol and an aqueous solution of cupric chloride with a ligand/metal ratio of 2. Heating was maintained for few minutes until dissolution of all ligand. Then a pinch of NaCl was added and the heating was continued. When the solution became clear, it was left to stand at room temperature. After a few days, green crystals were collected by filtration. They were dried over P_2_O_5_ in a desiccator for 48 h.

### Refinement

The methanol molecule is disordered across a centre of inversion. All O-bound H atoms were initially located in a difference map and refined with a O–H distance restraint of 0.84 (1) Å and an additional H···H restraint of 1.37 (2) Å for the water molecules. Later they were refined in the riding model with U_iso_(H) set to 1.5U_eq_(O). The C-bound H atoms were positioned geometrically [C-H = 0.93-0.96 Å] and refined using a riding model with U_iso_(H) = 1.2-1.5U_eq_(O). Reflections 110, 011 and 111 affected by beamstop were removed during refinement. The reflections 031, 313, 532 and 230 were omitted because the difference between their calculated and observed intensities are very large.

### Crystal data

**Table:**

[CuCl(C_9_H_12_N_4_)_2_]Cl·0.5CH_4_O·4H_2_O	*F*(000) = 1200
*M**_r_* = 574.98	*D*_x_ = 1.410 Mg m^−^^3^
Monoclinic, *P*2_1_/*c*	Mo *K*α radiation, λ = 0.71073 Å
Hall symbol: -P 2ybc	Cell parameters from 4291 reflections
*a* = 12.5213 (3) Å	θ = 2.6–29.8°
*b* = 15.5386 (4) Å	µ = 1.04 mm^−^^1^
*c* = 14.1806 (4) Å	*T* = 298 K
β = 100.883 (1)°	Block, green
*V* = 2709.40 (12) Å^3^	0.44 × 0.33 × 0.19 mm
*Z* = 4	

### Data collection

**Table:**

Bruker X8 APEXII area-detector diffractometer	7884 independent reflections
Radiation source: fine-focus sealed tube	5480 reflections with *I* > 2σ(*I*)
graphite	*R*_int_ = 0.029
φ and ω scans	θ_max_ = 30.0°, θ_min_ = 2.7°
Absorption correction: multi-scan (*SADABS*; Bruker, 2005)	*h* = −17→17
*T*_min_ = 0.668, *T*_max_ = 0.820	*k* = −20→21
47954 measured reflections	*l* = −19→19

### Refinement

**Table:**

Refinement on *F*^2^	Primary atom site location: structure-invariant direct methods
Least-squares matrix: full	Secondary atom site location: difference Fourier map
*R*[*F*^2^ > 2σ(*F*^2^)] = 0.038	Hydrogen site location: inferred from neighbouring sites
*wR*(*F*^2^) = 0.124	H-atom parameters constrained
*S* = 1.01	*w* = 1/[σ^2^(*F*_o_^2^) + (0.0649*P*)^2^ + 0.7437*P*] where *P* = (*F*_o_^2^ + 2*F*_c_^2^)/3
7884 reflections	(Δ/σ)_max_ = 0.001
323 parameters	Δρ_max_ = 0.39 e Å^−^^3^
0 restraints	Δρ_min_ = −0.26 e Å^−^^3^

### Special details

**Table:**

Geometry. All e.s.d.'s (except the e.s.d. in the dihedral angle between two l.s. planes) are estimated using the full covariance matrix. The cell e.s.d.'s are taken into account individually in the estimation of e.s.d.'s in distances, angles and torsion angles; correlations between e.s.d.'s in cell parameters are only used when they are defined by crystal symmetry. An approximate (isotropic) treatment of cell e.s.d.'s is used for estimating e.s.d.'s involving l.s. planes.
Refinement. Refinement of *F*^2^ against ALL reflections. The weighted *R*-factor *wR* and goodness of fit *S* are based on *F*^2^, conventional *R*-factors *R* are based on *F*, with *F* set to zero for negative *F*^2^. The threshold expression of *F*^2^ > σ(*F*^2^) is used only for calculating *R*-factors(gt) *etc*. and is not relevant to the choice of reflections for refinement. *R*-factors based on *F*^2^ are statistically about twice as large as those based on *F*, and *R*- factors based on ALL data will be even larger.

### Fractional atomic coordinates and isotropic or equivalent isotropic displacement parameters (Å^2^)

**Table:**

	*x*	*y*	*z*	*U*_iso_*/*U*_eq_	Occ. (<1)
Cu1	0.900612 (18)	0.233115 (15)	0.118071 (16)	0.04439 (9)	
Cl1	0.90806 (7)	0.08691 (4)	0.12169 (4)	0.0800 (2)	
N1	0.78145 (13)	0.23508 (10)	0.00716 (12)	0.0454 (3)	
N2	0.70109 (13)	0.28521 (11)	−0.13260 (12)	0.0477 (4)	
N3	1.05116 (12)	0.37246 (10)	0.01121 (11)	0.0417 (3)	
N4	0.96472 (12)	0.32276 (9)	0.02067 (10)	0.0393 (3)	
N5	1.01880 (13)	0.24643 (10)	0.22878 (12)	0.0450 (4)	
N6	1.09451 (12)	0.30513 (12)	0.36557 (12)	0.0486 (4)	
N7	0.74464 (12)	0.38061 (10)	0.21093 (10)	0.0399 (3)	
N8	0.83164 (11)	0.32983 (9)	0.20746 (10)	0.0379 (3)	
C1	0.68255 (17)	0.19476 (15)	−0.01803 (16)	0.0570 (5)	
H1	0.6547	0.1532	0.0179	0.068*	
C2	0.63247 (18)	0.22604 (15)	−0.10454 (17)	0.0571 (5)	
H2	0.5643	0.2102	−0.1384	0.068*	
C3	0.79083 (15)	0.28850 (11)	−0.06320 (12)	0.0403 (4)	
C4	0.89033 (15)	0.33733 (10)	−0.05888 (12)	0.0379 (3)	
C5	0.92968 (17)	0.39587 (12)	−0.11883 (13)	0.0452 (4)	
H5	0.8937	0.4163	−0.1780	0.054*	
C6	1.03251 (16)	0.41695 (11)	−0.07205 (13)	0.0443 (4)	
C7	0.6782 (2)	0.33629 (16)	−0.22015 (15)	0.0620 (6)	
H7A	0.6052	0.3252	−0.2531	0.093*	
H7B	0.7283	0.3211	−0.2609	0.093*	
H7C	0.6859	0.3963	−0.2042	0.093*	
C8	1.11508 (19)	0.47529 (14)	−0.10149 (17)	0.0588 (5)	
H8A	1.0878	0.4972	−0.1648	0.088*	
H8B	1.1811	0.4439	−0.1016	0.088*	
H8C	1.1295	0.5224	−0.0571	0.088*	
C9	1.14876 (18)	0.37281 (16)	0.08480 (18)	0.0626 (6)	
H9A	1.1915	0.4227	0.0771	0.094*	
H9B	1.1904	0.3218	0.0791	0.094*	
H9C	1.1290	0.3741	0.1470	0.094*	
C10	1.12060 (17)	0.21135 (15)	0.25698 (17)	0.0567 (5)	
H10	1.1517	0.1696	0.2236	0.068*	
C11	1.16802 (17)	0.24752 (15)	0.34115 (18)	0.0583 (5)	
H11	1.2372	0.2357	0.3759	0.070*	
C12	1.00555 (14)	0.30198 (12)	0.29551 (13)	0.0410 (4)	
C13	0.90411 (14)	0.34939 (11)	0.28650 (12)	0.0373 (3)	
C14	0.86365 (16)	0.41194 (12)	0.34037 (13)	0.0463 (4)	
H14	0.8983	0.4359	0.3982	0.056*	
C15	0.76163 (16)	0.43081 (12)	0.28999 (13)	0.0446 (4)	
C16	0.64923 (17)	0.37722 (16)	0.13561 (16)	0.0570 (5)	
H16A	0.6096	0.4302	0.1345	0.086*	
H16B	0.6038	0.3302	0.1474	0.086*	
H16C	0.6710	0.3690	0.0748	0.086*	
C17	0.6784 (2)	0.49353 (17)	0.31146 (19)	0.0672 (6)	
H17A	0.7067	0.5233	0.3702	0.101*	
H17B	0.6135	0.4631	0.3181	0.101*	
H17C	0.6618	0.5343	0.2599	0.101*	
C18	1.1111 (2)	0.35786 (17)	0.45289 (17)	0.0655 (6)	
H18A	1.1821	0.3467	0.4902	0.098*	
H18B	1.0569	0.3438	0.4900	0.098*	
H18C	1.1053	0.4176	0.4356	0.098*	
O5	0.6547 (5)	−0.0238 (4)	0.0811 (5)	0.130 (2)	0.50
H5A	0.6516	0.0148	0.1199	0.195*	0.50
C19	0.5593 (8)	−0.0440 (7)	0.0398 (6)	0.123 (3)	0.50
H19A	0.5173	0.0073	0.0231	0.185*	0.50
H19B	0.5258	−0.0785	0.0823	0.185*	0.50
H19C	0.5626	−0.0762	−0.0174	0.185*	0.50
O1	0.38934 (14)	0.50044 (13)	0.08744 (14)	0.0760 (5)	
H1A	0.3877	0.4702	0.0374	0.114*	
H1B	0.4383	0.5370	0.0841	0.114*	
O2	0.6051 (2)	0.73482 (18)	0.27534 (18)	0.1147 (9)	
H2A	0.6107	0.7026	0.2296	0.172*	
H2B	0.6581	0.7289	0.3199	0.172*	
O3	0.6009 (3)	0.6933 (2)	0.46268 (19)	0.1391 (11)	
H3A	0.5428	0.6909	0.4210	0.209*	
H3B	0.5919	0.7388	0.4938	0.209*	
O4	0.3845 (3)	0.4064 (2)	0.25444 (18)	0.1355 (10)	
H4A	0.3913	0.4417	0.2114	0.203*	
H4B	0.3836	0.3559	0.2350	0.203*	
Cl2	0.58501 (5)	0.62937 (4)	0.07857 (5)	0.07005 (17)	

### Atomic displacement parameters (Å^2^)

**Table:**

	*U*^11^	*U*^22^	*U*^33^	*U*^12^	*U*^13^	*U*^23^
Cu1	0.05202 (15)	0.03657 (13)	0.04530 (14)	−0.00040 (9)	0.01102 (10)	0.00375 (9)
Cl1	0.1459 (7)	0.0368 (3)	0.0565 (3)	0.0098 (3)	0.0171 (4)	0.0002 (2)
N1	0.0476 (9)	0.0430 (8)	0.0478 (8)	−0.0077 (7)	0.0150 (7)	−0.0046 (6)
N2	0.0480 (9)	0.0485 (9)	0.0462 (8)	0.0079 (7)	0.0078 (7)	−0.0106 (7)
N3	0.0440 (8)	0.0371 (7)	0.0465 (8)	−0.0030 (6)	0.0150 (6)	0.0028 (6)
N4	0.0412 (7)	0.0366 (7)	0.0423 (8)	−0.0017 (6)	0.0132 (6)	0.0050 (6)
N5	0.0451 (8)	0.0440 (8)	0.0481 (9)	0.0087 (6)	0.0147 (7)	0.0099 (7)
N6	0.0430 (9)	0.0481 (9)	0.0521 (9)	−0.0002 (7)	0.0025 (7)	0.0122 (7)
N7	0.0399 (8)	0.0383 (8)	0.0425 (7)	0.0049 (6)	0.0106 (6)	0.0043 (6)
N8	0.0379 (7)	0.0365 (7)	0.0405 (7)	0.0032 (6)	0.0101 (6)	0.0033 (6)
C1	0.0536 (12)	0.0569 (13)	0.0624 (13)	−0.0135 (10)	0.0162 (10)	−0.0089 (10)
C2	0.0450 (10)	0.0635 (14)	0.0624 (13)	−0.0067 (9)	0.0094 (9)	−0.0179 (10)
C3	0.0449 (9)	0.0368 (8)	0.0403 (9)	0.0034 (7)	0.0108 (7)	−0.0082 (7)
C4	0.0479 (9)	0.0322 (8)	0.0356 (8)	0.0038 (7)	0.0131 (7)	−0.0027 (6)
C5	0.0636 (12)	0.0383 (9)	0.0364 (8)	0.0039 (8)	0.0161 (8)	0.0031 (7)
C6	0.0598 (11)	0.0327 (8)	0.0463 (9)	0.0000 (7)	0.0248 (8)	0.0012 (7)
C7	0.0657 (14)	0.0666 (15)	0.0504 (11)	0.0174 (11)	0.0029 (10)	−0.0034 (10)
C8	0.0776 (15)	0.0408 (11)	0.0663 (13)	−0.0104 (9)	0.0347 (11)	0.0020 (9)
C9	0.0494 (12)	0.0639 (14)	0.0715 (14)	−0.0105 (10)	0.0036 (10)	0.0135 (11)
C10	0.0514 (11)	0.0585 (12)	0.0633 (13)	0.0173 (9)	0.0188 (10)	0.0174 (10)
C11	0.0438 (11)	0.0633 (13)	0.0666 (14)	0.0114 (9)	0.0073 (9)	0.0216 (11)
C12	0.0393 (9)	0.0391 (9)	0.0452 (9)	0.0011 (7)	0.0096 (7)	0.0129 (7)
C13	0.0418 (9)	0.0335 (8)	0.0373 (8)	−0.0018 (6)	0.0097 (7)	0.0066 (6)
C14	0.0544 (11)	0.0415 (10)	0.0427 (9)	0.0012 (8)	0.0084 (8)	−0.0016 (7)
C15	0.0533 (11)	0.0377 (9)	0.0459 (9)	0.0048 (7)	0.0172 (8)	0.0016 (7)
C16	0.0481 (11)	0.0620 (13)	0.0577 (12)	0.0108 (9)	0.0016 (9)	−0.0007 (10)
C17	0.0730 (15)	0.0591 (14)	0.0723 (15)	0.0229 (11)	0.0205 (12)	−0.0053 (11)
C18	0.0644 (14)	0.0619 (14)	0.0622 (13)	−0.0024 (11)	−0.0085 (11)	0.0021 (11)
O5	0.141 (5)	0.120 (5)	0.152 (5)	0.030 (4)	0.087 (4)	0.050 (4)
C19	0.127 (7)	0.145 (8)	0.113 (6)	0.004 (6)	0.060 (5)	0.030 (5)
O1	0.0805 (12)	0.0732 (11)	0.0803 (11)	−0.0101 (9)	0.0307 (9)	−0.0129 (10)
O2	0.139 (2)	0.123 (2)	0.0857 (16)	−0.0216 (16)	0.0300 (15)	−0.0303 (14)
O3	0.176 (3)	0.147 (3)	0.0957 (17)	0.028 (2)	0.0274 (18)	−0.0217 (18)
O4	0.195 (3)	0.115 (2)	0.0959 (17)	−0.022 (2)	0.0239 (18)	0.0127 (15)
Cl2	0.0692 (4)	0.0644 (4)	0.0750 (4)	−0.0088 (3)	0.0097 (3)	−0.0167 (3)

### Geometric parameters (Å, °)

**Table:**

Cu1—N1	1.9531 (17)	C8—H8B	0.96
Cu1—N5	1.9545 (17)	C8—H8C	0.96
Cu1—N4	2.2161 (14)	C9—H9A	0.96
Cu1—N8	2.2415 (14)	C9—H9B	0.96
Cu1—Cl1	2.2739 (6)	C9—H9C	0.96
N1—C3	1.320 (2)	C10—C11	1.351 (4)
N1—C1	1.374 (3)	C10—H10	0.93
N2—C3	1.348 (2)	C11—H11	0.93
N2—C2	1.368 (3)	C12—C13	1.453 (2)
N2—C7	1.456 (3)	C13—C14	1.390 (3)
N3—C6	1.350 (2)	C14—C15	1.372 (3)
N3—N4	1.357 (2)	C14—H14	0.93
N3—C9	1.450 (3)	C15—C17	1.499 (3)
N4—C4	1.340 (2)	C16—H16A	0.96
N5—C12	1.314 (3)	C16—H16B	0.96
N5—C10	1.374 (3)	C16—H16C	0.96
N6—C12	1.346 (2)	C17—H17A	0.96
N6—C11	1.374 (3)	C17—H17B	0.96
N6—C18	1.467 (3)	C17—H17C	0.96
N7—C15	1.349 (2)	C18—H18A	0.96
N7—N8	1.354 (2)	C18—H18B	0.96
N7—C16	1.445 (3)	C18—H18C	0.96
N8—C13	1.337 (2)	O5—C19	1.266 (10)
C1—C2	1.358 (3)	O5—H5A	0.82
C1—H1	0.93	C19—H19A	0.96
C2—H2	0.93	C19—H19B	0.96
C3—C4	1.450 (3)	C19—H19C	0.96
C4—C5	1.397 (2)	O1—H1A	0.85
C5—C6	1.372 (3)	O1—H1B	0.84
C5—H5	0.93	O2—H2A	0.83
C6—C8	1.493 (3)	O2—H2B	0.83
C7—H7A	0.96	O3—H3A	0.85
C7—H7B	0.96	O3—H3B	0.85
C7—H7C	0.96	O4—H4A	0.84
C8—H8A	0.96	O4—H4B	0.83
			
N1—Cu1—N5	173.03 (7)	C6—C8—H8B	109.5
N1—Cu1—N4	78.45 (6)	H8A—C8—H8B	109.5
N5—Cu1—N4	97.22 (6)	C6—C8—H8C	109.5
N1—Cu1—N8	97.33 (6)	H8A—C8—H8C	109.5
N5—Cu1—N8	77.82 (6)	H8B—C8—H8C	109.5
N4—Cu1—N8	98.90 (6)	N3—C9—H9A	109.5
N1—Cu1—Cl1	93.19 (5)	N3—C9—H9B	109.5
N5—Cu1—Cl1	93.78 (5)	H9A—C9—H9B	109.5
N4—Cu1—Cl1	128.60 (4)	N3—C9—H9C	109.5
N8—Cu1—Cl1	132.50 (4)	H9A—C9—H9C	109.5
C3—N1—C1	107.14 (17)	H9B—C9—H9C	109.5
C3—N1—Cu1	117.07 (13)	C11—C10—N5	108.7 (2)
C1—N1—Cu1	135.76 (15)	C11—C10—H10	125.6
C3—N2—C2	107.22 (17)	N5—C10—H10	125.6
C3—N2—C7	127.23 (19)	C10—C11—N6	106.85 (18)
C2—N2—C7	125.53 (19)	C10—C11—H11	126.6
C6—N3—N4	111.62 (15)	N6—C11—H11	126.6
C6—N3—C9	127.65 (16)	N5—C12—N6	110.84 (16)
N4—N3—C9	120.72 (15)	N5—C12—C13	119.79 (16)
C4—N4—N3	105.15 (14)	N6—C12—C13	129.37 (18)
C4—N4—Cu1	110.79 (11)	N8—C13—C14	111.07 (16)
N3—N4—Cu1	144.00 (11)	N8—C13—C12	113.71 (15)
C12—N5—C10	106.69 (18)	C14—C13—C12	135.21 (17)
C12—N5—Cu1	117.93 (12)	C15—C14—C13	105.27 (16)
C10—N5—Cu1	135.37 (15)	C15—C14—H14	127.4
C12—N6—C11	106.90 (18)	C13—C14—H14	127.4
C12—N6—C18	127.53 (18)	N7—C15—C14	107.11 (16)
C11—N6—C18	125.55 (18)	N7—C15—C17	122.55 (19)
C15—N7—N8	111.44 (15)	C14—C15—C17	130.34 (19)
C15—N7—C16	127.86 (16)	N7—C16—H16A	109.5
N8—N7—C16	120.70 (15)	N7—C16—H16B	109.5
C13—N8—N7	105.10 (14)	H16A—C16—H16B	109.5
C13—N8—Cu1	110.70 (11)	N7—C16—H16C	109.5
N7—N8—Cu1	144.10 (11)	H16A—C16—H16C	109.5
C2—C1—N1	108.2 (2)	H16B—C16—H16C	109.5
C2—C1—H1	125.9	C15—C17—H17A	109.5
N1—C1—H1	125.9	C15—C17—H17B	109.5
C1—C2—N2	107.16 (19)	H17A—C17—H17B	109.5
C1—C2—H2	126.4	C15—C17—H17C	109.5
N2—C2—H2	126.4	H17A—C17—H17C	109.5
N1—C3—N2	110.32 (17)	H17B—C17—H17C	109.5
N1—C3—C4	119.69 (16)	N6—C18—H18A	109.5
N2—C3—C4	129.94 (17)	N6—C18—H18B	109.5
N4—C4—C5	110.70 (16)	H18A—C18—H18B	109.5
N4—C4—C3	113.73 (15)	N6—C18—H18C	109.5
C5—C4—C3	135.56 (17)	H18A—C18—H18C	109.5
C6—C5—C4	105.54 (16)	H18B—C18—H18C	109.5
C6—C5—H5	127.2	C19—O5—H5A	109.5
C4—C5—H5	127.2	O5—C19—H19A	109.5
N3—C6—C5	106.99 (16)	O5—C19—H19B	109.5
N3—C6—C8	122.76 (19)	H19A—C19—H19B	109.5
C5—C6—C8	130.25 (18)	O5—C19—H19C	109.5
N2—C7—H7A	109.5	H19A—C19—H19C	109.5
N2—C7—H7B	109.5	H19B—C19—H19C	109.5
H7A—C7—H7B	109.5	H1A—O1—H1B	103.3
N2—C7—H7C	109.5	H2A—O2—H2B	110.7
H7A—C7—H7C	109.5	H3A—O3—H3B	102.6
H7B—C7—H7C	109.5	H4A—O4—H4B	111.9
C6—C8—H8A	109.5		
			
N4—Cu1—N1—C3	−4.61 (13)	C2—N2—C3—C4	176.81 (18)
N8—Cu1—N1—C3	93.03 (14)	C7—N2—C3—C4	−4.9 (3)
Cl1—Cu1—N1—C3	−133.39 (13)	N3—N4—C4—C5	0.24 (19)
N4—Cu1—N1—C1	177.6 (2)	Cu1—N4—C4—C5	178.19 (11)
N8—Cu1—N1—C1	−84.8 (2)	N3—N4—C4—C3	179.21 (14)
Cl1—Cu1—N1—C1	48.8 (2)	Cu1—N4—C4—C3	−2.84 (17)
C6—N3—N4—C4	−0.30 (19)	N1—C3—C4—N4	−0.8 (2)
C9—N3—N4—C4	−179.64 (18)	N2—C3—C4—N4	−178.02 (17)
C6—N3—N4—Cu1	−177.04 (15)	N1—C3—C4—C5	177.79 (19)
C9—N3—N4—Cu1	3.6 (3)	N2—C3—C4—C5	0.6 (3)
N1—Cu1—N4—C4	4.05 (12)	N4—C4—C5—C6	−0.1 (2)
N5—Cu1—N4—C4	−170.41 (12)	C3—C4—C5—C6	−178.75 (19)
N8—Cu1—N4—C4	−91.68 (12)	N4—N3—C6—C5	0.2 (2)
Cl1—Cu1—N4—C4	88.86 (12)	C9—N3—C6—C5	179.53 (19)
N1—Cu1—N4—N3	−179.3 (2)	N4—N3—C6—C8	−178.74 (17)
N5—Cu1—N4—N3	6.2 (2)	C9—N3—C6—C8	0.5 (3)
N8—Cu1—N4—N3	84.9 (2)	C4—C5—C6—N3	−0.1 (2)
Cl1—Cu1—N4—N3	−94.5 (2)	C4—C5—C6—C8	178.80 (19)
N4—Cu1—N5—C12	95.69 (14)	C12—N5—C10—C11	−0.5 (2)
N8—Cu1—N5—C12	−1.91 (13)	Cu1—N5—C10—C11	178.32 (15)
Cl1—Cu1—N5—C12	−134.62 (13)	N5—C10—C11—N6	0.4 (3)
N4—Cu1—N5—C10	−83.1 (2)	C12—N6—C11—C10	−0.1 (2)
N8—Cu1—N5—C10	179.3 (2)	C18—N6—C11—C10	178.7 (2)
Cl1—Cu1—N5—C10	46.61 (19)	C10—N5—C12—N6	0.5 (2)
C15—N7—N8—C13	−0.05 (19)	Cu1—N5—C12—N6	−178.63 (12)
C16—N7—N8—C13	179.66 (17)	C10—N5—C12—C13	−179.30 (16)
C15—N7—N8—Cu1	−175.72 (15)	Cu1—N5—C12—C13	1.6 (2)
C16—N7—N8—Cu1	4.0 (3)	C11—N6—C12—N5	−0.2 (2)
N1—Cu1—N8—C13	−172.90 (11)	C18—N6—C12—N5	−179.04 (19)
N5—Cu1—N8—C13	2.02 (11)	C11—N6—C12—C13	179.52 (18)
N4—Cu1—N8—C13	−93.51 (11)	C18—N6—C12—C13	0.7 (3)
Cl1—Cu1—N8—C13	85.92 (12)	N7—N8—C13—C14	0.25 (18)
N1—Cu1—N8—N7	2.63 (19)	Cu1—N8—C13—C14	177.54 (12)
N5—Cu1—N8—N7	177.6 (2)	N7—N8—C13—C12	−179.07 (13)
N4—Cu1—N8—N7	82.02 (19)	Cu1—N8—C13—C12	−1.78 (17)
Cl1—Cu1—N8—N7	−98.55 (19)	N5—C12—C13—N8	0.3 (2)
C3—N1—C1—C2	−0.7 (2)	N6—C12—C13—N8	−179.36 (17)
Cu1—N1—C1—C2	177.22 (15)	N5—C12—C13—C14	−178.76 (19)
N1—C1—C2—N2	0.4 (2)	N6—C12—C13—C14	1.5 (3)
C3—N2—C2—C1	0.1 (2)	N8—C13—C14—C15	−0.4 (2)
C7—N2—C2—C1	−178.17 (19)	C12—C13—C14—C15	178.76 (19)
C1—N1—C3—N2	0.8 (2)	N8—N7—C15—C14	−0.2 (2)
Cu1—N1—C3—N2	−177.57 (11)	C16—N7—C15—C14	−179.86 (19)
C1—N1—C3—C4	−176.90 (16)	N8—N7—C15—C17	179.76 (18)
Cu1—N1—C3—C4	4.7 (2)	C16—N7—C15—C17	0.1 (3)
C2—N2—C3—N1	−0.6 (2)	C13—C14—C15—N7	0.3 (2)
C7—N2—C3—N1	177.67 (18)	C13—C14—C15—C17	−179.6 (2)

### Hydrogen-bond geometry (Å, °)

**Table:**

*D*—H···*A*	*D*—H	H···*A*	*D*···*A*	*D*—H···*A*
O1—H1A···Cl2^i^	0.85	2.33	3.162 (2)	167
O1—H1B···Cl2	0.84	2.34	3.186 (2)	175
O2—H2A···Cl2	0.83	2.39	3.205 (3)	165
O3—H3B···Cl2^ii^	0.85	2.38	3.234 (3)	174
O4—H4A···O1	0.84	1.98	2.793 (3)	165
O4—H4B···O2^iii^	0.83	1.89	2.706 (4)	165
C11—H11···Cl2^iv^	0.93	2.75	3.592 (2)	151
C18—H18C···Cl1^v^	0.96	2.76	3.708 (3)	177

Symmetry codes: (i) −*x*+1, −*y*+1, −*z*; (ii) *x*, −*y*+3/2, *z*+1/2; (iii) −*x*+1, *y*−1/2, −*z*+1/2; (iv) −*x*+2, *y*−1/2, −*z*+1/2; (v) −*x*+2, *y*+1/2, −*z*+1/2.

## References

[bb1] Allen, C. F. H. & Wilson, B. D. (1963). US Patent No. 3 106 467.

[bb2] Bouhaddioui, S. (1993). Thèse de doctorat d’état, Université Mohammed 1er, Faculté des Sciences Oujda, Morocco.

[bb3] Bruker (2005). *APEX2*, *SAINT* and *SADABS* Bruker AXS Inc., Madison, Wisconsin, USA.

[bb4] El-Khawass, E. S. M. & Bistawroos, A. E. (1990). *Alex. J. Pharm. Sci.* **4**, 77–79.

[bb5] Farrugia, L. J. (1997). *J. Appl. Cryst.* **30**, 565.

[bb6] Farrugia, L. J. (1999). *J. Appl. Cryst.* **32**, 837–838.

[bb7] Hartfiel, U., Dorfmeister, G., Franke, H., Geisler, J., Johann, G. & Rees, R. (1993). Eur. Patent Appl. EP 542 388.

[bb8] Pearson, I. (1975). US Patent No. 3 883 549.

[bb9] Sheldrick, G. M. (2008). *Acta Cryst.* A**64**, 112–122. 10.1107/S0108767307043930 18156677

[bb10] Spek, A. L. (2009). *Acta Cryst* D**65**, 148–155. 10.1107/S090744490804362X PMC2631630 19171970

[bb11] Tjiou, E. M., Fruchier, A., Pellegerin, V. & Tarago, G. (1989). *J. Heterocycl. Chem.* **26**, 893–898.

[bb12] Trofimenko, S. (1993). *Chem. Rev.* **93**, 943–980.

[bb13] Tsuboi, S., Moriie, K., Hatsutori, Y., Wada, K., Sone, S., Oohigata, T. & Ito, A. (1994). Jpn Kokai Tokkyo Koho, JP 06 184 114.

